# Guidelines for the measurement of oxygen consumption rate in *Caenorhabditiselegans*

**DOI:** 10.1016/j.redox.2025.103723

**Published:** 2025-06-11

**Authors:** Anna Gioran, Niki Chondrogianni

**Affiliations:** Institute of Chemical Biology, National Hellenic Research Foundation, 11635, Athens, Greece

**Keywords:** Oxygen consumption rate, *C. elegans*, Mitochondria, Experimental protocol

## Abstract

Mitochondria are known as the powerhouse of the cell as through oxidative phosphorylation, they produce energy in the form of ATP. Nevertheless, mitochondria are also considered as the main producers of free radicals. Several mitochondrial parameters are needed to be examined to fully characterize mitochondria and the outcomes of their positive (i.e. energy production) or negative (i.e. production of free radicals/oxidative stress) function. Oxygen consumption rate (OCR) measurement is an excellent readout for mitochondrial respiratory capacity and it is the most frequently used assessment to examine mitochondrial function or as part of a broader bioenergetic profiling. Given the link between mitochondrial dysfunction, and increased oxidative stress and damage, and the fact that mitochondrial dysfunction is often reflected in OCR, its measurement is important for the complete characterization of the cellular redox status. Although much of this work is being done in cells or isolated mitochondria, there is an increasing need for the measurement of OCR in whole organismal models such as the nematode *Caenorhabditis elegans*. As a free-living organism with simple maintenance and conserved mitochondrial biology, *C. elegans* attracts interest as a model for ageing and age-related diseases, among others, in which bioenergetics but also various mitochondria-related redox aspects need to be evaluated. Therefore, the need for platforms suitable for OCR measurements in this model is evident. In this work, we have employed a newly developed system (Resipher) for the measurement of OCR in *C. elegans* and we outline basic protocols as well as the pharmacological interventions that can be used to assess the function of the respiratory chain. More specifically, we demonstrate the importance of the number of animals used in measurements that include mitochondrial complex inhibitors, how the presence of bacteria when used as a food source for the nematodes should be carefully considered and/or eliminated and how to avoid artefacts when measuring differently sized nematodes. The present work is not only intended to be used as a protocol for a specific measurement system but it can also be used as a guideline when setting up OCR experiments with any device, as it reveals parameters that may be overlooked and should be carefully considered.

## Highlights

• Protocol for oxygen consumption rate (OCR) measurements in *C. elegans* using Resipher system.
• Parameters to be considered in OCR measurements in *C. elegans* with any device.
• Animal number and body size, measurement duration and food source are important.

## 1 Introduction

Oxygen consumption rate (OCR) is an excellent readout for mitochondrial respiratory capacity and indeed it is the most frequently chosen parameter to identify mitochondrial dysfunction. Given that mitochondrial dysfunction has been clearly linked with several physiological processes such as ageing but also with disorders, including neurodegenerative diseases [1], OCR emerges as an important parameter. Furthermore, the effect of the metabolic rate on oxidative stress and ultimately on ageing and longevity has attracted a lot of interest. For a long time, it was believed that the rate of metabolism is proportionally linked to the production of Reactive Oxygen Species (ROS) and the enhancement of either was negatively correlated to longevity [2]. Nevertheless, this notion was challenged following studies in birds that, despite their high metabolic rate, exhibit enhanced longevity compared to mammals [3]. It was then shown that birds have a reduced rate of ROS production [4,5], further challenging the initial notion that high metabolic rate equals increased ROS production. Given how mounting evidence from various models suggests that metabolic rate and oxidative stress can be uncoupled [[6], [7], [8]], both mitochondrial respiration and ROS production should be measured to fully characterize the bioenergetic but also redox status of an individual.

Although we now have a more accurate picture of how metabolic rate does or does not affect ROS production, many of the related studies are being conducted in cells or isolated mitochondria. This is not surprising as most of the platforms for OCR measurements are optimized for cellular models and isolated mitochondria. Use of these platforms for small animals requires certain adjustments mainly due to the need for experimentation at different temperatures or the need of using mitochondrial inhibitors not commonly used in cells and isolated mitochondria.

*Caenorhabditis elegans* has many advantages over other models commonly used to study mitochondrial function. It allows assessments in an intact free-living organism, has short life cycle and lifespan, can be propagated fast and extensively with a low cost giving isogenic progeny, has a large variety of available mutants, while gene silencing through RNAi is straightforward [9]. Moreover, other mitochondrial parameters such as mitochondrial morphology [10] and mitochondrial membrane potential (MMP) [11] have been extensively studied in *C. elegans* that, when combined with OCR measurements, allow a wide mitochondrial characterization. Measuring OCR in *C. elegans* may be used as an ageing marker [12], healthspan indicator or longevity predictor [13], or even as a reliable readout for toxicity assessment [14]. Several sophisticated methods were employed in the past to measure OCR in *C. elegans* like optical probes [15] or microfluidic devices with oxygen-sensitive luminescent layers [16]. Nevertheless, approaches such as these, may require high expertise and this is why the simplicity offered by a system like the Seahorse XF Analyzer has been so appealing in measuring OCR in nematodes although it is more suitable for use in live cells. The rather extensive use of Seahorse XF Analyzers for OCR measurement in *C. elegans* has led to the development of acute response protocols to measure OCR following pharmacological manipulation of the electron transport chain (ETC) [17,18]. This has led to deep insights of the nematode ETC function that includes basal and maximal respiration, spare respiratory capacity (SRC), ATP turnover and proton leak.

In this paper, we outline several parameters that should be considered while measuring OCR in adult *C. elegans* and we provide detailed protocols and calculations on how the different components and functions of the respiratory chain can be assessed. Although several similar methods papers exist, they mostly use Seahorse XF Analyzers while we employed the Resipher system (Lucid Scientific, GA, USA), a newly developed and more affordable instrument for the measurement. Although we used this specific instrument for our measurements, all of the presented principles should be applied for any other system that measures OCR. Moreover, this work can be used as a guideline to standardize OCR measurements with different types of equipment in a more methodical way, thus being particularly helpful for novices in the field.

## 2 Materials and methods

### *2.1* *C. elegans* culture and preparation before OCR measurement

Animals were grown and maintained at 20 °C, using standard techniques for maintenance [19]. Wild type (wt) N2 (Bristol) animals were obtained from the Caenorhabditis Genetics Center (CGC). Day 1 gravid adults were used in all experiments. On the day of the measurement, the animals were transferred on a nematode growth medium (NGM) plate free of bacteria to crawl for a few minutes to get rid of traces of bacteria on their bodies. They were then transferred on NGM plates seeded with heat-inactivated (HI) *E. coli* OP50 (3 h at 60 °C) that had been concentrated 10 times via centrifugation. The animals were incubated on these plates for 1 h before being transferred into the wells of the dedicated 96-well plate for the measurement.

### 2.2 OCR measurements with complex inhibitors and carbonyl cyanide p-(trifluoromethoxy) phenylhydrazone (FCCP)

For the measurement of OCR, we used the Resipher system (Lucid Scientific, GA, USA). The wells intended for use were filled with X μL of M9 and worms fed with heat-inactivated (HI) OP50 were added. The number of animals per well varied and are reported for each experiment while at least 3 technical replicates were included in each experiment (see Results and Figure legends for details). Once all worms had been transferred into the wells, inhibitors or FCCP were added so that the volume of inhibitor/FCCP + the volume of M9 = 110 μL (final volume). FCCP (Fluorochem, #M04086), rotenone (Glentham Life Sciences, #GE1875) and oligomycin (Cell Signaling, #9996) were resuspended in DMSO at concentrations 1 mM, 250 μM and 20 μM, respectively. Antimycin A (Sigma, #A8674) was resuspended in 95 % ethanol at 50 mg/mL, NaN_3_ (Merck, #1.06688) was freshly prepared before each measurement and it was resuspended in M9 buffer at 200 mM to be used at a final concentration of 20 mM. In experiments with FCCP, oligomycin and rotenone, DMSO concentration was kept constant in all wells and ≤1 % to reduce toxicity [20]. In experiments with antimycin A, ethanol concentration was kept constant in all wells or NGM plates. In experiments with antimycin A added in the NGM plate, bacterial lawns were UV-inactivated (UVed) with a Vilber Lourmat Transilluminator (Vilber Lourmat GmbH, Germany; emitting 312 nm, for 10 min) prior to its addition. All measurements were conducted in an incubator at 20 °C. For the measurement of bacterial respiration, OP50 cultures were grown until saturation and split into 3 parts; the first part was kept at 4 °C until the measurement (live culture), the second part was incubated at 60 °C for 3 h (HI culture) and the third one was used to seed NGM plates. Once the lawns on the NGM plates had dried and grown (left overnight at room temperature), they were inactivated via UV light exposure. On the day of OCR measurement, 100 μL of live culture (brought to room temperature), 100 μL of HI culture and 100 μL of M9 in which UVed bacteria were resuspended were pipetted in the wells of a 96-well plate and OCR was measured.

#### 2.2.1 OCR calculations

OCR was calculated as follows.

• Basal respiration = Respiration in M9 wells - Respiration in NaN_3_ wells
• Maximal respiration = Respiration in FCCP wells - Respiration in NaN_3_ wells
• Spare respiratory capacity (SRC) = Maximal Respiration - Basal Respiration
• Non mitochondrial respiration = Respiration in NaN_3_ wells

At least 3 technical replicates per condition were measured in each experiment and the readings from each well were pooled for the calculations.

### 2.3 Brightfield imaging and size analysis of nematodes

Animals were loaded on a glass slide with a 2 % agarose pad and anesthetized with 25 mM tetramisole (Sigma, #L9756). A Leica TCS SPE confocal laser scanning microscope was used and the nematodes were imaged in the bright field mode with a 10x/0.25 objective. The length of the nematodes was measured in ImageJ.

### 2.4 Protein concentration determination in nematodes

For nematodes of different size, protein concentration was measured and used for the normalization of the OCR values. For short-term measurements (so that no larvae had hatched in the wells), nematodes were directly collected after the measurement by mixing the content of the well with M9 containing 0.1 % Triton to prevent nematodes from sticking on the tip walls. Nematode suspensions were left to settle in microcentrifuge tubes and washed 3 times with dH_2_O. Nematode pellets were then left overnight at −80 °C and the next day resuspended in 20–30 μL RIPA buffer supplemented with proteinase inhibitors and sonicated on ice until no intact nematodes were visible in the solution. Samples were centrifuged at 4 °C and 12000 rpm for 10 min and protein concentration was determined with DC (Bio-Rad, CA, USA, #500-0113, −0114, −0115) according to the manufacturer's instructions. For measurements over longer periods (more than ∼6 h) where larvae would be present in the wells after the end of the OCR measurement, the same number of animals used in the 96 well assay plate was collected separately in a microcentrifuge tube and protein concentration was measured there following the same procedure as above.

### 2.5 Statistical analysis

GraphPad Prism 10.1.2 (MA, USA) was used for the statistical analysis. Depending on the number of values of the compared groups a parametric (for >5 values) or a non-parametric (for ≤5 values) test was used with the appropriate post-hoc corrections when more than one groups were compared. All details for the statistical analysis used for each experiment are reported in the respective figure caption.

## 3 Results

### 3.1 Considerations on the number of nematodes used

Transferring nematodes one at a time in the wells of assay plates, or having expanded nematode populations enough for an OCR measurement, can be a painstaking and time-consuming process. OCR measurements might be necessary in nematodes that have been treated with expensive or hard to acquire compounds, in nematodes that carry mutations or transgenes that make them hard to propagate efficiently or even in aged animals. Therefore, we initially sought to determine the optimal number of adult nematodes that each well should contain to reliably detect differences between experimental conditions, while avoiding unnecessary population propagation or waste of valuable resources. OCR measurements with Seahorse XF Analyzers have been previously conducted with a large range of population sizes ranging from 10 [21] to 75 [22] adult nematodes per well while it has been argued that more than 25 animals per well may yield inaccurate readings [18]. Therefore, to establish the optimal number of nematodes for OCR measurements with Resipher, we ran a timelapse with 30, 40, 50 and 60 wt gravid adults (day 1 of adulthood) per well. Each population size produced a distinct trace showing OCR over time (Fig. 1A). Notably, negative readings might be produced during the initial measurements (up to 1.5 h from the beginning of the experiment) and this may vary from one experiment to the other (see Figure S1 and Fig. 2 for additional timelines). This is likely due to the time needed for the establishment of O_2_ gradients within the wells. Although the readings of the first 1 or 2 h might need to be excluded, plotting of the results per each time point showed that significant differences between the differently sized populations are already detectable 3 h after the initiation of the measurement and maintained up to 6 h (Fig. 1B and C).

**Fig. 1:**
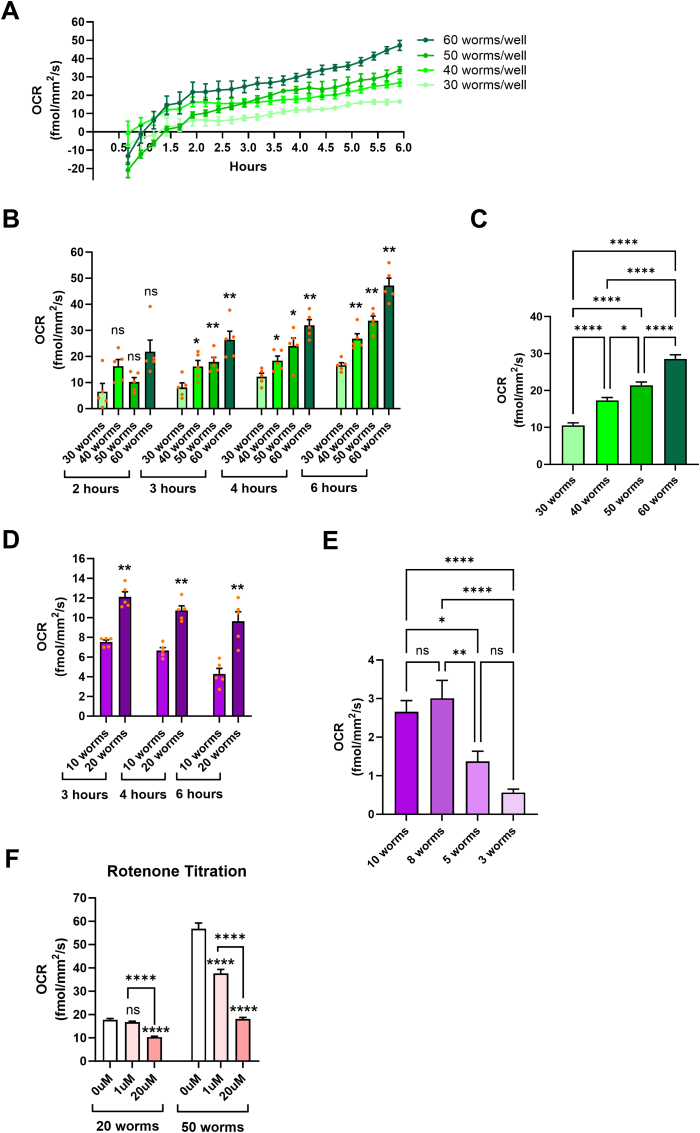
The effects of the number of nematodes used in OCR measurements. **(A)** 6-h timelapse of OCR measurements from wells containing 30, 40, 50 or 60 animals, respectively. The OCR readings from 5 wells/condition were pooled to plot the timelines. (**B**) OCR readings from panel A plotted in a bar chart on specific time points. Asterisks indicate the significance of differences between a given group and the immediately lower one in terms of used animals (i.e. 40 vs 30, 50 vs 40 and 60 vs 50), Mann Whitney test, ns-non significant, ∗p value < 0.05, ∗∗p value < 0.01. (**C**) Pooled OCR readings from panel A from the 4th hour of the timeline, Tukey's multiple comparisons test, ∗p value < 0.05, ∗∗∗∗p value < 0.0001. (**D**) Bar chart showing OCR for specific time points when 10 and 20 worms/well were used, 5 wells/condition, Mann Whitney test, ∗∗p value < 0.01. (**E**) Bar chart showing OCR from the 4th hour of the timeline when 10, 8, 5 or 3 worms/well were used, 5 wells/condition, Tukey's multiple comparison test, ns-non significant, ∗p value < 0.05, ∗∗p value < 0.01 ∗∗∗∗p value < 0.0001. (**F**) Bar chart showing responses to rotenone at the indicated concentrations. OCR readings from the 4th hour of the timeline are shown in this graph, Tukey's multiple comparison test, 4wells/condition, ns-non significant, ∗∗∗∗p value < 0.0001.

Although 6 h after the initiation of the experiment the differences between the various populations are still prominent (Fig. 1B), maintaining the worms in M9 buffer for 4–6 h without a food source is not recommended as prolonged starvation may cause metabolic changes [23], thus affecting the results. Additionally, when conducting experiments with gravid adults, one should also consider that hatching larvae may also affect the OCR measurements. Indeed, when we performed a long-term measurement we found that after ∼9 h the OCR started increasing for a few hours that coincided with hatching and ∼17 h after the initiation, it reached a plateau as many of the gravid adults died due to internal larvae hatching and the existing L1 larvae underwent arrest due to lack of food [23] (Fig. S1). To avoid these issues in case a long-term measurement is necessary, bacteria can be added in the assay well (e.g. concentrated OP50, see below recommendations on bacteria) while egg hatching should be prevented (e.g. with the use of 5-fluro-2ʹ-deoxyuridine -FUDR-).

To reveal what is the minimum number of animals giving a detectable OCR, we also used fewer than 30 worms per well. We found that the instrument could still discriminate between 10 and 20 worms per well giving small variation amongst technical replicates (Fig. 1D). We have also attempted to lower the sample to less than 10 worms per well; although we got some inconsistencies, we could still discriminate between wells containing 8, 5 or 3 animals (Fig. 1E). The observed high sensitivity should be taken into account in every experiment, as miscounting or accidental transfer of larvae inside the wells may interfere with the consistency of the values amongst technical replicates.

Although an experimental assay that requires only 10–20 or fewer worms per technical assay would be highly convenient, we sought to investigate whether such a low number of worms is appropriate to detect differences following pharmacological interventions such as exposure to mitochondrial inhibitors, a very common intervention when measuring OCR. To address this concern, we performed a titration with Rotenone, a widely used and selective complex I inhibitor, in wells containing 20 or 50 adult nematodes. We found that although 20 worms per well were sufficient to detect a decrease in OCR with 20 μM rotenone, the reduction could not be detected when 1 μM rotenone was used. On the contrary, with 50 worms per well, a statistically significant reduction in OCR was detectable even when nematodes were treated with as little as 1 μM rotenone, while the effect size was also pronounced (Fig. 1F).

In conclusion, our results suggest that even small population size differences may affect OCR readings significantly when using a Resipher system. Additionally, although we can have detectable OCR even with 3 animals, it is recommended that larger populations (ideally 50 animals) are used when examining the effects of mitochondrial inhibitors or other compounds to maximize sensitivity.

### 3.2 Assessing basal and maximal respiration and spare respiratory capacity (SRC)

Although complex IV is the only oxygen consumer within the ETC, certain pharmacological manipulations can isolate different respiratory states and facilitate assessment of the complete circuit, including basal respiration, proton (H+) leak and ATP turnover [24]. Although these assessments are typical when working with cellular models, OCR in *C. elegans* is mostly measured after uncoupling the mitochondria with FCCP or after inhibition of complex IV with NaN_3_ [18]. As shown in Fig. 2A, with the simple addition of FCCP (10 μM) and NaN_3_ (20 mM) directly in the assay well (without pre-treatment of the nematodes), basal mitochondrial respiration, SRC, maximal respiration and non-mitochondrial respiration can be measured.

**Fig. 2:**
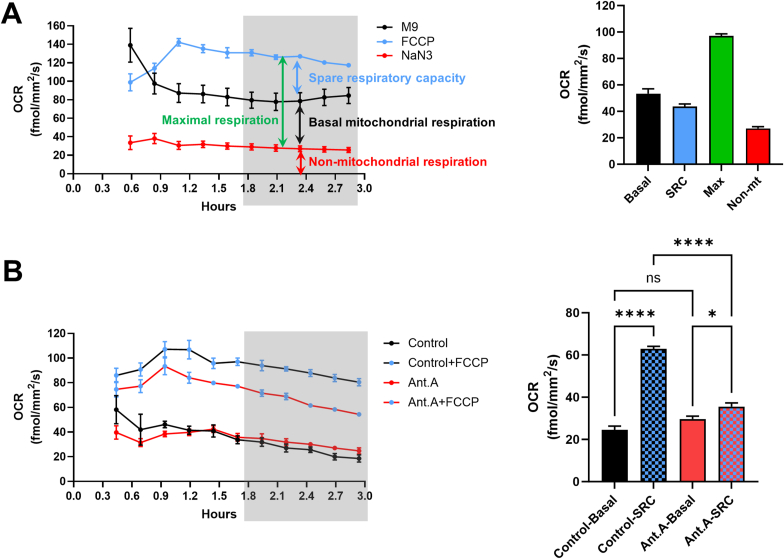
The effects of pharmacological interventions on the ETC. **(A)** Left panel: OCR measurement of animals treated with the uncoupler FCCP (10 μM) and the complex IV inhibitor NaN_3_ (20 mM) in a timeline of 3 h. OCR readings from 4 wells/condition were pooled to plot the timelines and 50 worms/well were used. The colored arrows show the values that are plotted in the right panel and the gray box indicates the time points the OCR readings of which were included in the right panel. (**B**) Left panel: OCR measurement of animals treated on the NGM plates for 24 h with vehicle (Control) or antimycin A (Ant. A; 0.1 μg/mL). OCR readings from 3 wells/condition were pooled to plot the timelines and 50 worms/well were used. The non-mitochondrial respiration has already been subtracted from the values shown on the timeline. The gray box indicates the time points the OCR readings of which were included in the right panel. Right panel: Bar chart showing OCR readings from left panel, Sidak's multiple comparison test, ns-non significant ∗p value < 0.05, ∗∗∗∗p value < 0.0001. (Basal = Basal mitochondrial respiration, SRC=Spare Respiratory Capacity, Max = Maximal respiration, Non mt = Non-mitochondrial respiration).

In experiments with cells, OCR is typically assessed after the addition of oligomycin (ATP synthase inhibitor), rotenone (complex I inhibitor) and antimycin A (complex III inhibitor). Nevertheless, no acute response in *C. elegans* has ever been reported so far for these compounds. We thus attempted to detect such responses. Even when we exposed the nematodes in the wells to the highest possible oligomycin and antimycin A concentrations (i.e. the highest we could reach before precipitation in the aqueous M9 buffer), we did not manage to generate an acute response (Fig. S2A). More specifically, oligomycin did not reduce OCR even after several hours of exposure. Notably, another ATP synthase inhibitor, namely dicyclohexylcarbodiimide (DCCD), has been shown to induce an acute OCR reduction in nematodes [17]. Antimycin A inhibited OCR increase over time only after ∼10 h of exposure (Fig. S2A), a non-optimal timeframe according to our findings (Fig. S1). Nevertheless, longer exposures to these inhibitors may have detectable effects. For example, incubating nematodes for 24 h prior to the OCR measurement on NGM plates with antimycin A, reduced their SRC although basal respiration remained unchanged (Fig. 2B). Finally, rotenone reduced OCR even after a short exposure (Fig. 1F, Fig. S2B for timeline) suggesting that it may be implemented in acute response protocols.

In conclusion, acute response protocols with FCCP, NaN_3_ and rotenone are feasible in nematodes and the relevant experiments can be conducted using the Resipher system.

### 3.3 Considerations when comparing OCR values between strains with different body sizes

Various mutations, especially the ones causing mitochondrial defects, as well as compound treatments may induce a smaller body size compared to wt or untreated counterparts. Smaller sized nematodes may display lower OCR values without necessarily meaning that they have a mitochondrial defect. Similarly, larger nematodes may show higher basal respiration values but this does not necessarily mean that they have enhanced mitochondrial function. To demonstrate one such example, we performed OCR measurements in wt and a transgenic strain overexpressing the proteasome subunit *pbs-5* (*pbs-5* OE) that was previously developed by our group [25]. Gravid nematodes of the *pbs-5* OE strain are slightly bigger compared to wt (control) animals of the same stage (Fig. 3A). When their basal respiration was measured it seemed that *pbs-5* OE animals respired more compare to wt (Fig. 3B). However, following normalization to the average length of each nematode population, the difference in the OCR between the two strains was abolished (Fig. 3C). Similarly, OCR normalization to protein content that is commonly used [24] also abolished the difference of basal respiration between control and *pbs-5* OE (Fig. 3D).

**Fig. 3:**
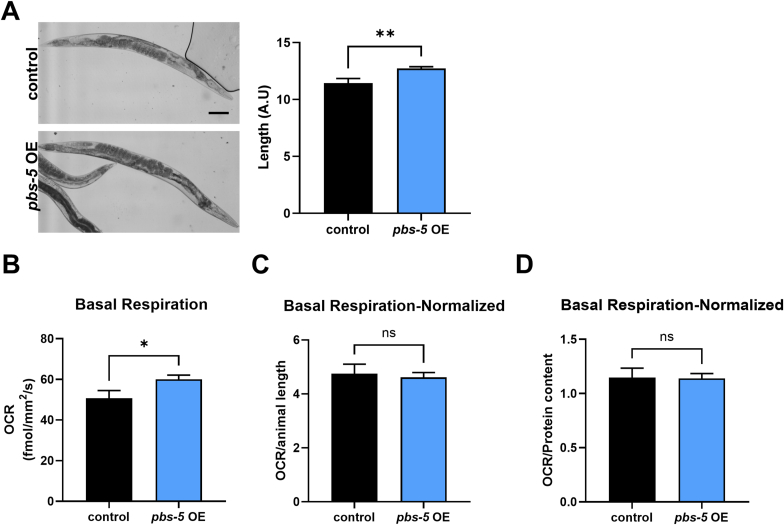
**OCR normalization to body size or protein content prevents artefacts**. (**A**) 15 control and 15 *pbs-5* OE animals were imaged and their length was quantified. Left panel shows representative images for each strain and the right panel shows animal length quantification. Scalebar shows 100 μm. Unpaired *t*-test, ∗∗p value < 0.01. (**B**) Basal respiration of control and *pbs-5* OE animals pooled from 4 wells/condition and from the 3rd hour of the measurement. 50 worms/well were used. Unpaired *t*-test, ∗p value < 0.05. (**C-D**) Basal respiration (as shown in panel B) normalized to animal length (C) and protein content (D). Unpaired *t*-test, ns-non significant.

In conclusion, comparing basal respiration amongst strains with different body sizes should include a normalization to size or protein content to exclude differences that come from varying sizes and not altered mitochondrial function.

### 3.4 Considerations regarding bacterial food sources used during OCR measurements

**Fig. 4:**
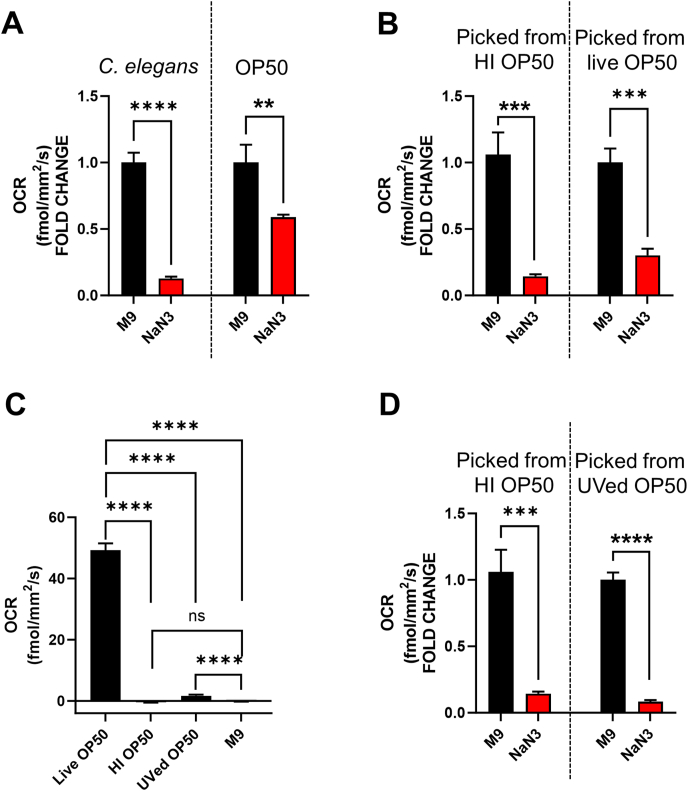
**Heat- or UV-inactivated bacteria do not interfere with OCR readings in nematodes.** (**A**) OCR fold change of nematodes (4 wells/condition, 50 worms/well) picked from plates seeded with HI OP50 that were left untreated (M9) or treated in the assay well with 20 mM NaN_3_ (left pair of bars). OCR fold change of 100 μL live OP50 culture (4 wells/condition) that was left untreated (M9) or treated in the assay well with 20 mM NaN_3_ (right pair of bars). For both data sets, the OCR readings from the 3rd hour of the timeline were used. Unpaired *t*-test, ∗∗p value < 0.001, ∗∗∗∗p value < 0.0001. (**B**) OCR fold change of nematodes picked from plates seeded with HI OP50 that were left untreated (M9) or treated in the assay well with 20 mM NaN_3_ (left pair of bars). OCR fold change of nematodes picked from plates seeded with live OP50 that were left untreated (M9) or treated in the assay well with 20 mM NaN_3_ (right pair of bars). Both sets of bars show pooled values for 3 wells for M9, 2 wells for NaN_3_, with 50 worms/well and the OCR readings from the 4th hour of the timeline. Unpaired *t*-test, ∗∗∗p value < 0.0005. (**C**) Bar chart showing OCR of live or dead (HI or UVed) OP50. Sterile M9 buffer was used as a reference (3 wells/condition, OCR reading taken from the 4th hour of the timeline. Asterisks indicate separate unpaired t-tests, ns-non significant, ∗∗∗∗p value < 0.0001. (**D**) OCR fold change of nematodes picked from NGM plates seeded with HI OP50 that were left untreated (M9) or treated in the assay well with 20 mM NaN_3_ (left pair of bars). OCR fold change of nematodes picked from plates seeded with UVed OP50 that were left untreated (M9) or treated in the assay well with 20 mM NaN_3_ (right pair of bars). Both sets of bars show pooled values for 3 wells for M9, 2 wells for NaN_3_, with 50 worms/well and the OCR readings from the 4th hour of the timeline. Unpaired *t*-test, ∗∗∗p value < 0.0005.

In this study, we have measured OCR in nematodes in bacteria-free M9 buffer. Although this is a valid approach for short-term measurements, experiments that require OCR measurements for longer periods or during development should include a food source, as starvation will result in developmental arrest or internal egg hatching (bagging) that will eventually kill off the population [23]. However, even traces of live bacteria may interfere with the OCR measurements of the nematodes in various ways. Firstly, bacteria proliferate and their OCR continues to increase for many hours (Fig. S3) as opposed to nematodes that mostly reach a plateau after several hours (Fig. S1). Secondly, live bacteria may not respond to mitochondrial inhibitors in the same manner with the nematodes, creating background OCR after the addition of the inhibitor. For example, when treated with 20 mM NaN_3_, nematodes kept in bacteria-free M9 reduced their respiration by almost 90 %, whereas a bacterial suspension exposed to the same concentration of NaN_3_ at the same time point, reduced its respiration by less than 50 % (Fig. 4A). To assess the effects of traces of live bacteria on OCR measurements, we picked nematodes from an NGM plate seeded with live bacteria leading to the presence of traces of live bacteria in the wells as well as nematodes from an NGM plate seeded with HI OP50. Although the animals that were fed HI OP50 for 1 h before the measurement showed ∼85 % reduction of OCR in the presence of NaN_3_, nematodes that were fed live OP50 showed only ∼70 % of OCR reduction (Fig. 4B). Although in both cases the reduction is statistically significant, it is clear that the presence of even traces of live bacteria interferes with the response to a complex IV inhibitor. Taken together, these data suggest that the presence of bacteria may lead to overestimation of the non-mitochondrial respiration of the nematodes. This issue should be carefully considered when using other inhibitors or compounds that need to be investigated for their effects on the OCR of nematodes.

Based on the above, we recommend the use of dead bacteria for the measurement of OCR in nematodes. Although for the purposes of this study we used HI bacteria, other methods of killing the bacteria can be used such as antibiotics [14], paraformaldehyde [26] or UV radiation. As shown in Fig. 4C, HI bacteria have an OCR that is indistinguishable from the bacteria-free M9 buffer, but UVed bacteria have some residual respiration. However, in the presence of NaN_3_, nematodes picked from a plate with UVed bacteria reduce their respiration by ∼90 % similarly to what was found in nematodes picked from plates seeded with HI bacteria (Fig. 4D), suggesting that UVed bacteria are also appropriate for OCR experiments.

In conclusion, the presence of bacteria even in traces in the suspension containing the nematodes that are intended for OCR measurements, may affect the OCR readings and create background. If a food source is necessary to facilitate long-term OCR measurements, then it is highly recommended that dead bacteria are used.

## 4 Discussion

OCR is an important mitochondrial parameter that needs to be measured to fully characterize mitochondria and to identify mitochondrial dysfunction. Resipher is a newly developed device for the measurement of OCR. It has already been successfully used in many studies using mouse myoblasts [27], human endothelial and smooth muscle cells [28], mouse T cells [29], in algae-laden bioprinted constructs [30], in placenta barrier on-a-chip models [31] but also in *C. elegans* [32]. Our study however, is the first to give detailed technical guidance on the use of the device in *C. elegans*. We specifically used adult nematodes and identified various parameters that should be considered when using Resipher for OCR measurements in nematodes. More specifically, the numbers of the animals used in the presence and absence of inhibitors should be tested before performing experiments. Only dead bacteria should be used if the experimental set up necessitates a food source (i.e. for long-term experiments or developmental studies). When measuring differently sized nematodes (due to treatments or compounds), OCR readings should be normalized to animal size or protein content. Although our work was performed with the Resipher system, the suggested considerations should be applied with any OCR analyzer when nematodes are the model of interest.

Resipher is a handheld device that can be placed in any incubator and function at temperatures between 20 °C and 45 °C making it ideal for *C. elegans* that is, in most cases, kept at 20 °C. This temperature flexibility makes Resipher a potentially useful tool for other non-cellular models such as the marine flatworm *Macrostomum lignano* [33] or *Danio rerio* (zebrafish) [34] that require 20 °C and 28.5 °C, respectively. Our work may also serve as a guideline to establish experiments in nematodes, including experimentation in larvae that has been already performed with other analyzers [13,14,18].

One of the drawbacks of Resipher is that the system does not offer an automated injection of compounds during the measurement. In other words, the sensor lid has to be removed to add the compounds manually and then the lid has to be replaced in its position. This means that if at least 1 h is required before O_2_ gradients are established in the wells, every compound addition will require at least 1 h before useable readings can be obtained. Although this is not an issue when using Resipher for long-term experiments, for short-term measurements it is problematic. To overcome this issue, we simply started with multiple wells per condition and added FCCP or inhibitors from the beginning of the experiment. Although this does not allow monitoring of the responses to mitochondrial function-modifying compounds in one specific well over time, we found that inhibitors worked in a similar fashion from one biological replicate to another, making this approach acceptable. A second drawback of this system is the lack of a concomitant measurement of extracellular acidification rate (ECAR) which may be a useful readout when working with cells, but it seems to have no value in experiments with *C. elegans* [35].

Existing analyzers have their own unbeatable advantages but also drawbacks. This study does not aim to highlight which analyzer is better but rather to show which parameters should be taken into account when measuring OCR in *C. elegans*, irrespectively of the system used. Moreover, this study aims to show that valuable results can be produced in *C. elegans* even with a budget-friendly system such as Resipher which is important for groups that cannot afford a highly sophisticated but also expensive analyzer. Overall, Resipher presents a promising tool for measuring OCR in nematodes and careful consideration of experimental parameters such as animal number, inhibitor usage, normalization method and food source is crucial for accurate results.

## CRediT authorship contribution statement

**Anna Gioran:** Writing – review & editing, Writing – original draft, Visualization, Methodology, Investigation, Funding acquisition, Formal analysis, Conceptualization. **Niki Chondrogianni:** Writing – review & editing, Writing – original draft, Visualization, Validation, Supervision, Resources, Project administration, Funding acquisition, Conceptualization.

## Data availability

All data are available in the main text or the supplementary materials. The datasets generated during and/or analyzed during the current study are available from the corresponding author on reasonable request.

## Declaration of competing interest

None.

## Figures and Tables

**figs1:**
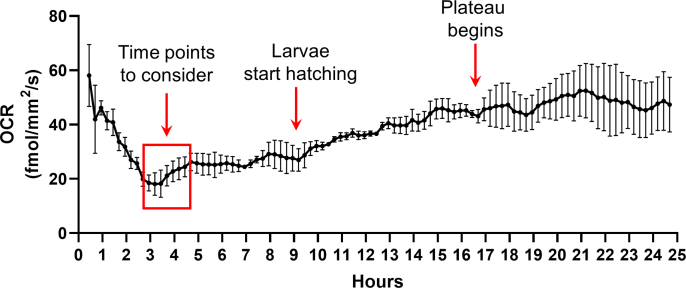


**figs2:**
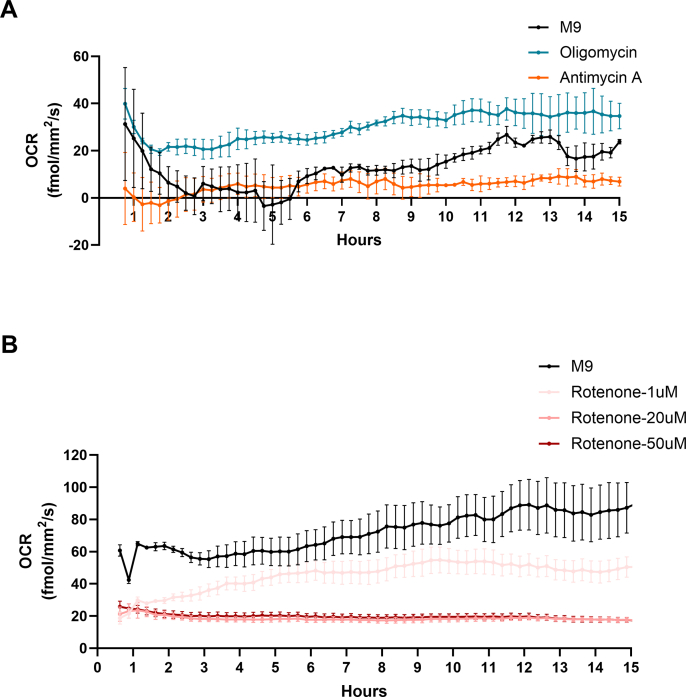


**figs3:**
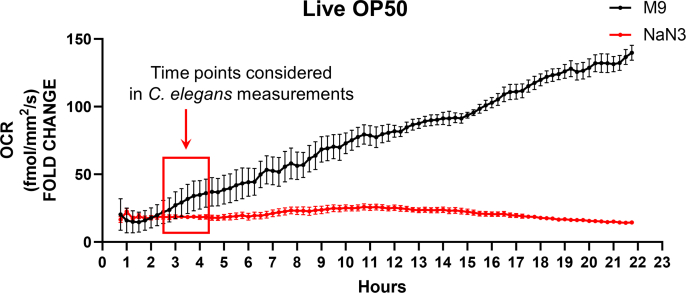


## Data Availability

Data will be made available on request.
