# Supplementary material for: Guidelines for the measurement of oxygen consumption rate in Caenorhabditiselegans
Source: Redox Biol. 2025 Jun 11;85:103723. doi: 10.1016/j.redox.2025.103723 (PMC12205836; doi:10.1016/j.redox.2025.103723)
Supplement: Multimedia component 1 [file mmc1.docx]

**Supplementary Figure Captions**

**Figure S1. OCR over time.** OCR readings from a 25-hour long timeline, readings were pooled from 3 wells and 50 worms were used per well.

**Figure S2. Acute responses are not induced by all commonly used mitochondrial complex inhibitors in *C. elegans*.** (**A**) OCR readings from a 15-hour long timeline in nematodes left untreated (M9) or treated with high concentrations of oligomycin (340.5 μg/mL) and antimycin A (227 μg/mL) in the assay wells. Each line shows pooled OCR readings from 3 wells and 50 worms/well were used. (**B**) OCR readings from a 15-hour long timeline in nematodes left untreated (M9) or treated with rotenone at the depicted concentrations in the assay wells. Each line shows pooled OCR readings from 4 wells and 50 worms/well were used.

**Figure S3. OCR of live OP50 is increased over time.** OCR readings from a 22-hour long timeline of 100 μl of live OP50 culture that was left untreated (M9) or treated with 20 mM NaN_3_ in the assay well. The lines represent pooled values from 4 wells/condition.
